# T cell avidity and tumor recognition: implications and therapeutic strategies

**DOI:** 10.1186/1479-5876-3-35

**Published:** 2005-09-20

**Authors:** Mark D McKee, Jeffrey J Roszkowski, Michael I Nishimura

**Affiliations:** 1 Department of Surgery, The University of Chicago, Chicago, IL, USA

## Abstract

In the last two decades, great advances have been made studying the immune response to human tumors. The identification of protein antigens from cancer cells and better techniques for eliciting antigen specific T cell responses *in vitro *and *in vivo *have led to improved understanding of tumor recognition by T cells. Yet, much remains to be learned about the intricate details of T cell – tumor cell interactions. Though the strength of interaction between T cell and target is thought to be a key factor influencing the T cell response, investigations of T cell avidity, T cell receptor (TCR) affinity for peptide-MHC complex, and the recognition of peptide on antigen presenting targets or tumor cells reveal complex relationships. Coincident with these investigations, therapeutic strategies have been developed to enhance tumor recognition using antigens with altered peptide structures and T cells modified by the introduction of new antigen binding receptor molecules. The profound effects of these strategies on T cell – tumor interactions and the clinical implications of these effects are of interest to both scientists and clinicians. In recent years, the focus of much of our work has been the avidity and effector characteristics of tumor reactive T cells. Here we review concepts and current results in the field, and the implications of therapeutic strategies using altered antigens and altered effector T cells.

## T cell – tumor antigen interactions

### Antigens recognized by tumor reactive T cells

One of the key advances in the study of tumor immunology has been the identification of specific protein antigens recognized by tumor reactive T cells. Both MHC class I and MHC class II-restricted peptides have been identified from tumor-associated antigens (TAA) on a variety of human cancers. The identification of TAA has dramatically improved our ability to study the interactions between tumor reactive T cells and their targets, and has been the foundation of new clinical strategies to treat cancer patients [1-4].

TAA can be classified into five groups based on their origin, structure, and tissue expression. Several of the earliest identified TAA were melanoma-melanocyte differentiation antigens [5-7]. These antigens, such as MART-1, gp100, and tyrosinase, are expressed exclusively by cells of the melanocyte lineage. They are considered to be shared TAA because they are expressed by the vast majority of melanomas tested [5-10]. A second group of antigens called cancer/testis antigens are expressed by normal testis and a variety of human tumors including cells from melanoma, breast, bladder, colon, lung, head and neck, gastric, ovarian, neuroblastoma, and prostate cancers [11-14]. These antigens are not universally expressed by tumors of a particular histology, but instead are seen in only a small fraction of any tumor type [15-22]. A third group of antigens are derived form normal viral proteins, and are found exclusively on tumors that are induced by viral infection of human cells [11-13]. This category includes antigens such as EBNA-3 on Epstein Barr virus-induced lymphomas and the E6 and E7 proteins on human papilloma virus-induced cervical cancers [23-25]. The fourth group of antigens is characterized by aberrant expression in tumors relative to normal tissues [12,13]. Many of these proteins have been implicated in tumorigenesis or tumor growth and progression. Antigens such as Her-2/neu and p53, each of which may be highly over-expressed by tumor cells relative to normal tissues, fall into this category [26-32]. The final group of antigens is characterized by protein structures that contain mutations in the sequence [12,13]. These mutations alter the processing, presentation, or recognition of the epitope by the immune system. Such mutations have been described for the β-catenin and CDK4 genes, as well as others [25,33,34]. With the wide variety of antigens available for recognition by the immune system, it is not surprising that proteins expressed by many common tumors can be targeted by T cells.

To date, tumor reactive T cells have been identified that recognize dozens to hundreds of different peptide epitopes. Epitopes may be presented by MHC class I for CD8 T cell recognition, or by class II molecules for CD4 T cell recognition. Epitopes for TAA restricted by HLA A, B, C, and DR alleles have been identified [11-13]. Epitopes with the most clinical relevance are those that are restricted by the most common MHC molecules (HLA-A2, C7, A1, B44, A3, B7, and DR4). These epitopes can be targeted in treatments for the greatest number of patients [35].

### T cell avidity and tumor cell recognition

Avidity describes the strength of interaction between a T cell and its target antigen. Avidity is usually measured via T cell activation by a target cell, and is a sum of several contributing components, such as T cell receptor (TCR) expression levels, TCR/peptide/MHC binding affinity, co-stimulatory molecule expression, and the extracellular microenvironment. Experimental evidence suggests that avidity may exert fine control over the response of an activated T cell by influencing the binding and signaling of TCR complexes on the T cell surface. Certain T cell responses are extremely sensitive to activation by antigen. It has been reported that one TCR/peptide/MHC interaction can lead to activation of a T cell as measured by Ca^+2 ^mobilization, three interactions lead to target cell lysis, and ten interactions lead to full activation as measured by T cell proliferation [36]. However, other more commonly used methods for measuring T cell function, such as cytokine secretion or cytolysis, fail to detect T cell responses unless far more peptide is encountered on the target. These assays are commonly performed using peptide loaded antigen presenting cells (APC) as targets in co-culture with T cells. The avidity of a T cell population can be defined by the concentration of antigen required to elicit a T cell response after target loading. In these assays, a high avidity T cell requires less antigen (< 1 nM peptide loaded on an APC) for activation than a moderate (1–100 nM peptide loaded on an APC) or low (>100 nM peptide loaded on an APC) avidity T cell [37].

Many investigators have demonstrated a correlation between T cell avidity and target recognition of T cell populations that recognize virally infected targets, murine tumor models, and human cancers. The first reported relationship between T cell avidity and target cell recognition examined interactions between polyclonal T cell populations and the protein antigen gp160 on HIV infected target cells [38]. In this study, immunization with high doses of antigen led to expansion of T cells with low avidity, whereas immunization with low doses of antigen led to expansion of T cells with high avidity. In a second report, it was shown that if high avidity T cells were exposed to high levels of antigen on targets, activation induced T cell death resulted [39]. These studies illustrated that T cell avidity plays an important role in both T cell priming and T cell response to antigen. Zeh et al. subsequently examined whether T cell avidity also influenced recognition of antigens expressed by tumor cells using a murine melanoma model [4]. In this study, high avidity T cells were raised to the antigens TRP-2 or p15E by stimulating T cells with very low amounts of antigenic peptide. In adoptive therapy experiments, the resultant high avidity T cells were more effective at eliminating lung metastases from B16 melanoma than low avidity T cells. Similar results have been seen with human T cells. Dudley et al. examined the response of individual T cell clones that recognized the melanoma TAA gp100:209–217 [40]. In co-culture with targets, the peptide load required for response by individual T cell clonotypes varied by several logs. Furthermore, there was a correlation between the relative avidity of the T cell clonotypes and their ability to recognize tumor cells. Taken together, these mouse and human results suggest that the relative sensitivity of a T cell to antigen influences its ability to recognize tumors, and that high avidity T cells are required for efficient anti-tumor immunity.

Though it is intuitive that high avidity T cells would better recognize tumors than low avidity T cells, there are reports of T cell populations which do not follow these avidity rules. Many T cells have been raised for TAA recognition through stimulation of naïve lymphocytes by peptides selected according to known MHC binding motifs [41]. The 369–376 peptide from Her-2/neu has generated conflicting reports regarding the relationship between T cell avidity and tumor target recognition. Several groups have identified T cells that recognize Her-2/neu:369–376 peptide as well as Her-2/neu^+ ^tumor cells [26,27,42-44]. However, others have identified high avidity T cells that recognize peptide loaded targets but not tumors. Two clinical trials of immunization with the Her-2/neu:369–376 peptide resulted in the detection of T cells reactive with peptide loaded cells but not tumor cells [45,46]. These contrasting results suggest that the relationship between avidity and target recognition *in vivo *is complex, and that it is likely under the influence of other significant factors. Identifying and controlling these other factors may be vital if T cells with the genetic capacity and sufficient avidity to recognize TAA are to function as potent anti-tumor effectors.

### Experimental studies of TCR affinity and T cell avidity

TCR affinity is the strength of the molecular interaction between the receptor and peptide-MHC complex. TCR affinity has been proposed by some as the single most important component of T cell avidity, which is in agreement with current models of T cell activation that are based on the stability of TCR/peptide/MHC contact. However, experimental evidence can be found both supporting and opposing this point of view. For example, several groups have reported that bright tetramer staining, and thus high affinity TCR/peptide/MHC binding, correlates with high avidity T cell-target interactions [47,48]. On the other hand, other groups have found no correlation between tetramer binding and T cell avidity [49,50]. In several investigations, we have evaluated the avidity and affinity of T cells and TCR based on 1) recognition of APC's loaded with low concentrations of peptide, 2) recognition of tumor targets, or 3) an ability to signal without CD8 coreceptor binding. These studies, the results of which are detailed below, have shown that T cells with identical receptors may behave with different avidities in different circumstances.

T cell clones with identical TCR's may have different relative avidity for peptide loaded APC targets. T cell clones that recognize the HLA-A2 restricted TAA epitope gp100:209–217 were isolated from patients with malignant melanoma. DNA sequence analysis of the TCR subunits was performed on the clones, and several clones with identical receptors (sister clones) were identified. Assays measuring cytokine secretion by the sister clones after stimulation with peptide loaded APC targets or melanoma tumor targets revealed different relative avidities and differing abilities to recognize various tumor lines. These observations are not confined to melanoma reactive T cells or human TCR. Sister T cell clones recognizing the Her-2/neu:369–377 peptide have been isolated with different reactivities against the same Her-2/neu expressing target cells, and studies in animal models have also found T cells sharing the same TCR that have markedly different avidities [51].

High avidity T cells may have receptors that bind peptide-MHC complex with low affinity. A gp100:209–217 reactive T cell clone (R6C12) isolated from a patient with malignant melanoma was shown to have extremely high avidity and recognize HLA-A2^+ ^gp100 positive tumor cells [52,53]. Despite the high avidity of the R6C12 cells, they stained poorly with gp100:209–217 tetramers, suggesting that they had low affinity receptors. Tetramer staining by these cells was enhanced using a modified gp100 peptide that more tightly bound the HLA-A2 molecule [54]. Binding of modified tetramers was easily inhibited by anti-CD8 mAb, providing further evidence that despite the high avidity of CTL clone R6C12, its TCR had relatively low affinity. We have used gene transfer studies to characterize the R6C12 TCR in more detail [55,56]. The R6C12 receptor was cloned, and the receptor was transferred to Jurkat cells using a retroviral construct. These cells, derived from a human T cell lymphoma, do not express the CD8 coreceptor. Transduced Jurkat cells recognized peptide antigen on loaded APC targets with high avidity yet failed to recognize tumor cells, suggesting that the affinity of the receptor for peptide-MHC was insufficient for T cell signaling without coreceptor binding. Subsequently, the R6C12 TCR was transferred to peripheral blood T cells from normal donors [57]. These cultures, in contrast to transduced Jurkat cells, demonstrated the high avidity of the original R6C12 T cell clone. In sum, these data showed that the high avidity of the R6C12 T cell was not due to a high affinity TCR.

Finally, low avidity T cells may have receptors that exhibit characteristics of high affinity TCR/peptide/MHC binding. We have described a tyrosinase reactive T cell with low-moderate avidity characteristics in assays using peptide loaded APC targets, but with the high affinity TCR characteristic of CD8 independence. Of note, this T cell is also capable of recognizing tumor cell targets. A T cell clone recognizing a HLA-A2 restricted epitope from tyrosinase was isolated from the CD4+ population of a patient with malignant melanoma, and the receptor was used for TCR transfer studies like those described above. Both the original human T cell clone and transduced murine 58α-β-cells, which lack human CD8, were able to recognize HLA-A2^+ ^tyrosinase^+ ^tumor cells, even though greater than 100 ng/ml of peptide on targets was required to stimulate IL-2 secretion in APC co-culture assays. In direct contrast to the R6C12 TCR described above, this TCR from a low avidity T cell clone binds and signals in the absence of CD8 coreceptor. Taken together, our studies suggest to us that T cell avidity does not necessarily predict the affinity of the TCR, and that T cells are likely able to modulate their avidity independent of TCR affinity.

### Other factors influencing T cell recognition of targets

If T cells have the capacity to alter their antigen responsiveness by factors independent of their antigen receptor, molecular mechanisms other than the TCR must be implicated. Investigations by others have described numerous mechanisms by which T cell function may be altered in cancer patients. Mizoguchi et al. reported that T cells from mice bearing MCA 38 colon carcinoma tumors had reduced expression of CD3ζ chain expression on their surface, and that they had reduced levels of the tyrosine kinases p56^*lck *^and p59^*fyn *^[58]. Given that CD3ζ chain, p56^*lck *^and p59^*fyn *^are required for TCR-mediated signaling to occur [59], decreased expression of these molecules in tumor bearing hosts will result in impairment of T cell immunity. It was recently reported that the levels of L-arginine in the cell culture medium could regulate CD3ζ chain expression [60] and that the enzyme arginase I produced by macrophages may regulate the levels of L-arginine in cancer patients [61]. Other investigators have shown that tumor bearing mice have lower levels of the transcription factor NFκB [62]. These signaling defects have been confirmed in several mouse tumor models and in patients with colorectal carcinoma, renal cell cancer, head and neck cancers, and other malignancies [63-67]. Other metabolic pathways also appear to regulate T cell function, such as oxidative stress from hydrogen peroxide released by cells of the monocyte/macrophage lineage [68] and the level of tryptophan metabolites resulting from indoleamine 2,3-dioxygenase expression by macrophages [69,70]. Clearly, the influence of tumors on the physiology of the host may impact the ability to mount an immune response to malignancy by myriad mechanisms.

The CD8 coreceptor and its influence on the recognition of T cell targets deserve special emphasis. The CD8 coreceptor plays a critical role in the activation of some CD8^+ ^T cells by binding to the α3 domain of MHC class I and recruiting the kinase p56^*lck *^to the CD3 complex [71]. As discussed above, the dependence upon CD8 coreceptor function by a specific T cell clone is greatly influenced by the TCR/peptide/MHC binding characteristics of the cell. Classically, CD8 is described as a T cell membrane αβ heterodimer [72,73]. Recently, a CD8 αα homodimer form has been described [74]. Transfection studies have shown that the CD8 αβ heterodimer has higher affinity for MHC class I and p56^*lck *^than the CD8 αα homodimer, and that the αβ heterodimer more efficiently mediates T cell activation [74]. The ratio of CD8 αβ to CD8 αα as well as the ability for CD8 αβ to co-localize with the TCR to lipid rafts can have a profound impact on T cell avidity [51]. Future investigations will further clarify the role of coreceptor molecules in T cell tumor recognition, and may lead to new immunotherapy strategies based in part on T cell coreceptor function.

## Enhancing tumor recognition with modified TAA

### Enhancing the immunogenicity of TAA by enhancing MHC-peptide binding

Tumor antigen based clinical trials have led to relatively few clinical responses [75-80]. In addition, many cancer vaccine trials show little evidence of anti-tumor immunity in the peripheral blood of patients following vaccination [78,81]. In an effort to enhance the immunogenicity of known tumor antigens, investigators have introduced modifications into the amino acid sequences of known epitopes. Amino acid substitutions at MHC anchor positions in the antigenic peptide can lead to enhanced peptide/MHC binding [82], and can enhance the immunogenicity of an otherwise weakly immunogenic peptide both *in vitro *and *in vivo *[83-86]. The melanoma epitope gp100:209-217-2M is a well-studied example of an anchor residue-substituted peptide. Substituting a methionine for the native threonine at position 2 enhances binding of this peptide to HLA-A2 9-fold. More importantly, this M substitution enhances the immunogenicity of the peptide *in vitro *and *in vivo *with the resulting T cells having the capacity to recognize tumor cells [75,83].

Modifications of weakly immunogenic peptides at MHC anchor residues can result in other desirable effects, such as enhancing a peptide's stability in solution. The stability of the weakly immunogenic HLA-A2 restricted peptide antigen NY-ESO-1:155–163 is enhanced by an amino acid substitution at an MHC anchor residue [82]. A substitution of valine for cysteine at position 9 in the peptide not only enhances binding to HLA-A2, but also prevents disulfide bridge formation, thus eliminating dimerization of the peptide in solution [85]. Similarly, a substitution of a serine or alanine for the cysteine at position 2 of the HLA-A1 restricted tyrosinase:243–251 decreases the amount of peptide required to elicit T cell responses *in vitro *by two to three logs [87]. This simple approach of modifying the MHC binding residues of weakly antigenic peptides represents a powerful strategy for activating T cell populations that would otherwise be unresponsive to stimulation by the native antigen.

### Enhancing the immunogenicity of TAA by altering TCR contact residues

It has been shown that immunization with xenogeneic proteins can lead to enhanced immunity to the native protein. The genes encoding the human or rodent homologs of several tumor antigens have been used to vaccinate mice [41,88-90]. In these studies, the xenogeneic antigens routinely resulted in greater immune responses, leading to improved anti-tumor immunity. It was speculated that differences in the amino acid sequence between the xenogeneic antigen and the target antigen resulted in heteroclitic peptides (peptide analogs substituted at positions other than MHC contact residues) that were capable of inducing both effector and helper T cell responses. This hypothesis was directly tested using a peptide from the murine tumor antigen AH-1 [91]. Substituting an alanine for a valine at position 5 increased the binding to the TCR while having no impact on binding to the murine MHC I molecule. This substitution increased the ability of the AH-1 peptide to elicit CTL responses that protect mice from challenges with AH-1 expressing tumors [91]. These animal studies indicated that modifications to TCR contact residues can enhance the immunogenicity of peptide antigens.

Several investigations have also examined the response of human T cells to peptides modified at TCR contact residues [92-94]. One such study identified a heteroclitic peptide for the immunodominant HLA-A2 restricted epitope from human carcinoembryonic antigen, CEA:605–613. Substituting an aspartic acid for the asparagine at position 6, a TCR contact residue, enhances the capacity of this peptide to elicit CEA reactive T cells that can recognize CEA antigen on tumor cells [92]. Furthermore, clinical responses have been reported in colon cancer patients receiving a tumor vaccine comprised of autologous dendritic cells loaded with this heteroclitic CEA peptide [95]. Based on these promising results, other groups have evaluated modified peptides and identified heteroclitic peptides from several tumor antigens [82,94,96]. These modified peptides represent a promising approach for vaccinating cancer patients with otherwise weakly immunogenic antigens.

### Influence of peptide modifications on the T cell repertoire

Despite the ability of modified peptides to elicit strong anti-tumor immune responses when used for vaccinating patients, these peptides have generally failed to induce effective anti-tumor immunity and tumor regression [75,77]. Among several possible explanations for these results, one must consider whether modified peptides will optimally stimulate the TAA reactive T cell repertoire *in vivo*. The T cell repertoire has tremendous diversity due in part to the structure of the TCR molecule. TCR α and β chains consists of a variable (V) segment, a joining (J) segment, and a constant (C) region with the β chain also containing a diversity (D) region. Germline rearrangements occurring within the TCR α and β loci during T cell development randomly join different V-J or V-D-J regions into a single transcriptional unit. The majority of the TCR diversity is the result of the random insertion or deletion of nucleotides at the junctions between the V and J segments for the α chain, and between the V and D and the D and J segments for the β chain. It is these V-J and V-D-J junctions of the α and β chains respectively that encode the putative third complementarity determining region (CDR3), the structural feature of the TCR critical for antigen recognition [97,98].

Though initial reports suggested that there was a limited TCR repertoire used by tumor reactive T cells [99-104], we and others have failed to find evidence of restricted TCR V gene usage [105-112]. When we performed a detailed analysis of the TCR V genes used by MART-1:27–35 and gp100:209–217 reactive T cells, we found that 19 (out of a possible 46) different TCR Vβ were used by the MART-1:27–35 reactive T cell clones [105,108,110,113-116], and 16 different TCR Vβ were used by gp100:209–217 reactive T cell clones (unpublished). Further, no homology was found within the CDR3 regions of the TCR β chains of MART-1:27–35 or gp100:209–217 reactive T cell clones. These observations suggest that there is likely to be considerable TCR diversity among tumor reactive T cells.

Amino acid substitutions in peptides at the TCR contact residues can influence TCR binding and alter the TCR repertoire. This was elegantly demonstrated in a study using single TCR chain transgenic mice. Animals expressing the transgene for a single TCR subunit chain on all T cells were vaccinated with the native moth cytochrome C (MCC) peptides or peptides containing non-conservative amino acid substitutions at the TCR contact residues. MCC reactive T cell hybridomas were isolated from the T cell repertoire after vaccination. By introducing a positively charged amino acid residue into the immunizing peptide, the investigators could induce the presence of negatively charged amino acids in the non-transgenic TCR chains of reactive clones [117]. Thus, alterations in the immunizing peptide influenced the animals' T cell repertoire significantly. We have seen similar changes in the TCR repertoire of patients after vaccination with peptide antigens modified at MHC anchor residues. We found that after vaccination with a gp100:209–217 peptide containing methionine instead of a threonine at position 2, T cell clones could be isolated from patients that recognized the modified peptide but not the native peptide or tumor cells [118]. One patient was identified from whom gp100:209 specific tumor reactive T cell clones could be isolated prior to vaccination. After vaccination, none of the peptide reactive T cell clones isolated from his peripheral blood were able to recognize tumor cells. These results indicate that even changes in the antigenic peptide which do not face the TCR can impact on the TCR repertoire. Given these observations, the potential effects on the T cell repertoire must be considered when contemplating vaccine strategies using substituted peptides.

## Enhancing tumor recognition by modifying T cells

### Generating tumor reactive T cell populations by TCR transfer

Generating an effective anti-tumor response *in vivo *requires the presence of T cell precursors capable of recognizing TAA. In many cancer patients, TAA reactive precursors can not be expanded from harvested tumor tissue, lymphoid tissue, or peripheral blood samples. It is not clear whether this is due to the low frequency of T cells against self-antigens, which comprise the majority of shared TAA, or due to an inability to activate or induce proliferation of reactive cells *in vitro*. A potential solution for these patients is to engineer tumor reactive T cells from naïve lymphocytes using gene therapy techniques. The validity of this approach has been established in pre-clinical studies briefly described above: through the use of specially designed DNA constructs, gene modification of effector T cells *in vitro *has enabled investigators to re-direct the specificity of T cell populations and T cell clones toward TAA. The majority of work in this area has used single chain antibody constructs bound to intracellular T cell signaling domains, although several investigators have transferred naturally occurring two-chain TCR molecules with their associated activities.

Redirecting T cell specificity through TCR gene therapy requires the transfer of naturally occurring TCR α and β chains to alternate effectors. TCR gene therapy has potential advantages over other adoptive immunotherapy strategies, such as the relative uniformity of the therapeutic agent and the precision with which the transduced T cell population can be measured before and after treatment. The feasibility of redirecting T cell specificity by TCR gene transfer was demonstrated by Dembic *et al*. in 1986 [119]. With the identification of the first shared tumor antigens for human melanoma in the early 1990's [120], we set out to transfer TAA recognition to a naïve lymphocyte population using this strategy. A TCR recognizing the melanoma antigenic peptide MART-1:27–35 was chosen for initial studies, since MART-1 is expressed by most melanomas and the epitope is restricted by the predominant MHC allele expressed in the United States, HLA-A2. The unique TCR α and β chain sequences from two HLA-A2 restricted, MART-1/Melan A reactive T cell clones were identified [105]. The Jurkat cell line was co-transfected with plasmids containing the α and β chain genes, and transfected cells were cloned in limiting dilution. Expression of the introduced TCR was confirmed, and the functional capacity of transfected clones with varied levels of TCR expression was determined by co-culturing the transduced population with peptide loaded target cells. Transfected Jurkat clones secreted IL-2 in response to culture with MART-1 loaded targets but not targets loaded with an irrelevant peptide. Furthermore, the functional avidity of the transfected clones correlated with the expression level of the transferred TCR. This was the first demonstration that a TAA specific TCR could be transferred with its characteristic antigen recognition to alternate T cells. Since these studies, Jurkat cells have been used to evaluate the transfer of other TCR's, including an HLA-A1 restricted TCR specific for MAGE-3 [121,122].

Next, we attempted to transfer the MART-1:27–35 reactive TCR to primary human T cells from peripheral blood [123,124]. A retroviral vector, designated A7, was constructed for transducing lymphocytes with the MART-1 receptor. To facilitate incorporation of retrovirus into the target cell genome, peripheral blood lymphocytes (PBL) were stimulated to proliferate with anti-CD3 antibody and IL-2 [125]. Transduced primary T cells were able to recognize peptide loaded targets as well as HLA-A2+ melanoma cells. Clones generated from these cultures had varied effector functions in response to co-culture with target cells. Further analysis revealed that only those that expressed the CD8 coreceptor were capable of recognizing tumor cells. Clones which expressed only the CD4 coreceptor could only recognize targets loaded with an excess of exogenous peptide, suggesting that the transferred receptor was dependent upon CD8 for full receptor function. This study verified that T cells suitable for adoptive immunotherapy could be re-directed to recognize tumor cells by TCR gene transfer. TCR's specific for a number of TAA and viral antigens associated with tumor development have been successfully introduced into T cells via retroviral gene transfer. These include TCR's specific for melanoma antigens MAGE-3, gp100, tyrosinase and CAMEL, the widely expressed oncoprotein MDM2, and the Epstein-Barr Virus protein LMP2 expressed by Hodgkin's lymphoma [57,121,122,126-130]. Recently, the transfer of a TCR into T cells with known specificity has been shown to result in individual cells reactive to both antigens [131,132]. It is therefore conceivable to engineer individual T cells with the ability to recognize multiple TAA.

The two-chain approach to TCR transfer has been modified by other investigators to address inherent problems of the approach with TCR subunit expression and pairing. When full-length TCR genes are introduced into normal T cells the native TCR α and β chains may pair with the exogenous TCR β and α chains respectively. This serves to dilute the number of functionally paired TCR's on the cell surface [133,134], and it raises the possibility that TCR's with unknown specificity could be generated, possibly leading to unexpected autoimmunity. To counter these problems, chimeric TCR genes have been generated by fusing the cytoplasmic signaling domain of CD3ζ to MAGE-1 reactive TCR α and β genes [135]. The chimeric TCR gene successfully conferred MAGE-1 reactive function to T cells following retroviral transfer. Notably, subunit genes were shown to pair exclusively to each other following retroviral transfer to T cells, preventing both dilution of functional transferred TCR and generation of TCR's with unknown specificity.

### Viral vectors for TCR transfer

Several viral vectors have been investigated for human gene therapy. Adenoviruses were the first viral vectors used due to their abilities to infect both dividing and non-dividing cells and to generate very high titer viral stocks. However, adenoviral vectors lack the ability to provide long-term transgene expression and are highly immunogenic. The viral vector of choice for many gene therapy studies, particularly in haematopoietic cells, is the retrovirus. Retroviruses infect only dividing cells and incorporate into the host cell genome, resulting in long-term transgene expression. They have low immunogenicity, providing a combination of beneficial properties for their use in gene therapies. Removal of the structural genes (*gag*), gene encoding enzymes for nucleic acid metabolism (*pol*), and the envelope encoding genes (*env*) serves to prevent self replication of the retrovirus following infection of target cells. The transgene TCR subunits and, commonly, a gene for cell selection encoding antibiotic resistance or a cell surface marker are then inserted under the control of the LTR and internal promoters. Our laboratory now employs a vector in which segments of the LTR have been replaced with elements of the cytomegalovirus (CMV) immediate early gene promoter. This hybrid promoter allows higher transcription levels in packaging cells leading to higher retroviral titer. We use both an internal promoter and IRES to allow for transcription of TCR genes and a selectable marker [136]. Other retroviruses that have been used for transfer of genes to human cells include murine stem-cell viruses and lentiviruses. Lentiviruses are a subset of retroviruses that are more genetically complex than MMLV. Their low immunogenic properties coupled with the capability of infecting non-dividing cells have made them a candidate for use in gene therapy. Recently, several groups have demonstrated lentiviral based gene transfer to primary human T cells [137-140]. While transduction of non-dividing T cells is possible, it has been repeatedly shown that T cell activation is still necessary for high level transfer and expression of the transgene. Furthermore, while use of retroviral based gene therapy is clinically established, lentiviral based therapies are not yet approved for clinical use.

### Generating tumor reactive T cell populations with chimeric antibody-receptors

Chimeric antibody receptors are another single chain alternative to TCR for redirecting T cell specificity to TAA. Chimeric immunoglobulin (cIg) receptors are composed of the heavy and light chain variable regions of an antibody fused to the transmembrane/intracellular portion of a lymphocyte signaling molecule. The most commonly used transmembrane/intracellular portions are from the Fc εRI-γ chain and the CD3-ζ chain. cIg receptors, described shortly after the development of single chain Ab molecules in the 1980's, are attractive constructs for modifying T cell specificity because their binding is not MHC-restricted, and because cIg can recognize intact surface proteins without the need for antigen processing and presentation by the target cell [141]. TCR transduced T cells, on the other hand, are more likely to demonstrate normal antigen binding and signaling behavior, which may be important for eliciting optimal CTL responses.

In 1993, Stancovski *et al*. reported anti Her-2/neu activity by T cell hybridomas transduced with Her-2/neu specific cIg fused to the Fc εRI-γ chain [142]. Subsequent studies by other investigators have demonstrated the efficacy of cIg receptor constructs specific for the breast cancer antigens Her3 and Her4 [143,144]. Ovarian cancer, lung cancer, melanoma, prostate cancer, and renal cell carcinoma are among the tumors that have been targeted with cIg receptor retroviral constructs by various groups [145-149]. Several groups have targeted glycoprotein molecules such as carcinoembryonic antigen (CEA) and GA733-2 that are expressed by a majority of colorectal cancers and other tumors of gastro-intestinal origin, and their cIg transduced T cells have shown efficacy *in vitro *and in murine models [150-154]. A comparison of CEA-directed cIg fused to the Fc εRI-γ chain or the CD3-ζ chain found that despite similar levels of transgene expression, CD3-ζ-linked cIg were able to better control the growth of CEA-expressing tumors in murine models [155]. Recently, these constructs have been further engineered to incorporate a costimulatory signaling mechanism [156-159]. Constructs containing the heavy and light chain variable regions of an antibody, the CD28 signaling domain, and the CD3-ζ chain in series were first described by Finney *et al *in 1998. T cells transduced with cIg containing the CD28 signaling domain have shown enhanced ability to control the growth of CEA-expressing tumors in murine models [158,160].

In summary, techniques for altering T cell-tumor interactions through gene transfer are being widely investigated. At the present time, several groups of investigators are addressing the methodologic and regulatory hurdles that must be overcome in preparing these agents for clinical use. The first clinical trials of TCR gene therapy have recently been initiated. If promising, the early scientific and clinical results of these studies may soon stimulate broad interest in TCR gene therapy for cancer and associated areas of investigation.

## Conclusion

Despite the wealth of information that has been acquired pertaining to T cell recognition of tumors, we are left with far more questions than answers regarding ways by which the immune response might be manipulated to improve cancer treatment. Though the T cell repertoire is expansive, the repertoire of tumor reactive cells in any individual may be very limited, or may be difficult to activate and expand either *in vitro *or *in vivo*. The relationship between TCR affinity, T cell avidity, and T cell effector function is complex. This may account for the disparity between our success in stimulating antigen reactive precursor T cells through immunization and generating cells for adoptive therapy *in vitro*, and our inability to achieve a high rate of durable clinical responses. A universal approach to immunization against tumor antigens or adoptive immunotherapy may not be possible for any tumor type. Instead, combined therapeutic approaches or therapy optimized for the individual may be necessary. Current and future investigations of specific T cell – tumor interactions and novel therapeutics will determine whether broadly effective immune therapies are to be realized.

## Competing interests

The author(s) declare that they have no competing interests.

## Authors' contributions

MDM conceived the design and organization for the review, participated in the immunological research included, and drafted the manuscript. JJR performed the immunological research included and participated in drafting the manuscript. MIN conceived, designed, and coordinated the immunological research included and participated in drafting the manuscript. All authors read and approved the final manuscript.
